# Small Animal Models for Human Metapneumovirus: Cotton Rat is More Permissive than Hamster and Mouse

**DOI:** 10.3390/pathogens3030633

**Published:** 2014-07-24

**Authors:** Yu Zhang, Stefan Niewiesk, Jianrong Li

**Affiliations:** Department of Veterinary Biosciences, College of Veterinary Medicine, The Ohio State University, Columbus, OH 43210, USA; E-Mails: Zhang.707@osu.edu (Y.Z.); Niewiesk.1@osu.edu (S.N.)

**Keywords:** human metapneumovirus, animal model, BALB/c mice, Syrian golden hamsters, cotton rats

## Abstract

Human metapneumovirus (hMPV) is the second most prevalent causative agent of pediatric respiratory infections worldwide. Currently, there are no vaccines or antiviral drugs against this virus. One of the major hurdles in hMPV research is the difficulty to identify a robust small animal model to accurately evaluate the efficacy and safety of vaccines and therapeutics. In this study, we compared the replication and pathogenesis of hMPV in BALB/c mice, Syrian golden hamsters, and cotton rats. It was found that BALB/c mice are not permissive for hMPV infection despite the use of a high dose (6.5 log_10 _PFU) of virus for intranasal inoculation. In hamsters, hMPV replicated efficiently in nasal turbinates but demonstrated only limited replication in lungs. In cotton rats, hMPV replicated efficiently in both nasal turbinate and lung when intranasally administered with three different doses (4, 5, and 6 log_10_ PFU) of hMPV. Lungs of cotton rats infected by hMPV developed interstitial pneumonia with mononuclear cells infiltrates and increased lumen exudation. By immunohistochemistry, viral antigens were detected at the luminal surfaces of the bronchial epithelial cells in lungs. Vaccination of cotton rats with hMPV completely protected upper and lower respiratory tract from wildtype challenge. The immunization also elicited elevated serum neutralizing antibody. Collectively, these results demonstrated that cotton rat is a robust small animal model for hMPV infection.

## 1. Introduction

Human metapneumovirus (hMPV) is a newly discovered paramyxovirus, first identified in 2001 in the Netherlands in infants and children experiencing respiratory tract infections similar to the symptoms caused by human respiratory syncytial virus (hRSV) [1]. Soon after its discovery, hMPV was recognized as a globally prevalent pathogen. Epidemiological studies suggest that 5%–15% of all respiratory tract infections in infants and young children are caused by hMPV, a proportion second only to that of human respiratory syncytial virus (hRSV). Virtually all children by the age of 5 were seropositive [1,2,3,4,5,6]. HMPV causes upper and lower respiratory tract infection with a spectrum of illnesses that range from asymptomatic infection to severe bronchiolitis. Infections in young adults usually cause mild flu-like symptoms. However, the infection can cause more severe disease in infants, children, the elderly, and immunocompromised individuals. HMPV infections in the upper respiratory tract are often associated with symptoms such as cough, fever, and rhinorrhea. In rare cases, infection can cause conjunctivitis, diarrhea, vomiting, and rash. HMPV infections in the lower respiratory tract can cause pneumonia, bronchiolitis, croup, and asthma exacerbation [7]. These clinical signs caused by hMPV are similar to and usually indistinguishable from hRSV and PIV3. In addition, co-infection with hRSV has been observed in 5%–17% of patients infected with hMPV. However, it is not clear if co-infection is related to the exacerbated disease. Despite major efforts, there are no therapeutics or vaccines available for hMPV [7,8].

A major hurdle for hMPV vaccine development and antiviral drug discovery is the difficulty of identifying a suitable animal model. An animal model that supports viral replication in its respiratory tract and faithfully reproduces human disease is instrumental in elucidating the mechanism of viral pathogenesis, nature of protective immunity, and development of humoral and cellular immune responses during infection. It could also be useful to accurately evaluate the efficacy and safety of vaccines and therapeutics. Since the first discovery of hMPV in 2001, several small animal models (such as mice, cotton rats, hamsters, guinea pigs, and ferrets) and non-human primate models (such as chimpanzees, rhesus macaques, and African green monkeys) have been reported for the study of virus-host interaction, viral pathogenesis, and antiviral immunity. To date, all these animal models do not mimic the signs of human disease except for chimpanzees. However, the degree of hMPV replication in the upper and lower respiratory tract in these animal models is variable. Because of the accessibility and availability of reagents and tools, most studies have been focused on mouse, hamster, and cotton rat models. In fact, many contradictory results have been reported for these animal models. Therefore, it is necessary to re-evaluate the permissiveness of animal models for hMPV infection.

Mice are the most commonly used animal model in biomedical research field because of its cost-effectiveness, and the availability of a vast number of inbred strains and reagents. BALB/c mice have been used to evaluate live attenuated vaccine candidates for hRSV. However, the usefulness of the mouse model was confounded by the fact that the replication of hRSV in mice model is limited [9,10,11]. Previously, it has been reported that hMPV infection in BALB/c mice leads to signs of illness, including weight loss, ruffled coat, huddling and respiratory clinical symptoms (such as heavy breathing) [12,13]. Interestingly, Alvarez *et al.* reported biphasic growth kinetics for hMPV (strain hMPV/CAN98-75) in lungs of BALB/c mice in which peak titers occurred at days 7 and 14 postinfection, and infectious hMPV was persistent in lungs up to day 60 postinfection [12]. Specifically, hMPV reached peak virus titers at days 7 postinfection (8 log_10 _PFU/g lung tissue) and declined to 5.8 log_10_ PFU/g of lung tissue at day 10 postinfection, followed by a second peak virus titer at day 14 postinfection (7 log_10 _PFU/g lung tissue). This biphasic replication kinetics has not been seen in other animal models including non-human primates, hamsters, cotton rats, and other mouse species. In addition, this unusual high viral replication has not been observed for any of the other paramyxoviruses (such as RSV). In fact, several groups reported that hMPV replication in different inbred strains of mice are highly restricted in the upper and lower respiratory tract [14,15]. In one study, hMPV/TN96-12 replication ranges from non-detectable to 2.93 log_10_ PFU/g in mouse lungs depending on the mouse strain [15]. In another study where BALB/c mice were intranasally inoculated with 5 log_10_ PFU of hMPV/NL/1/00, the yield was 2.40 log_10_ PFU/g lung tissue [14].

Syrian golden hamsters (*Mesocricetus auratus*) previously have been used to evaluate the efficacy of vaccine candidates of human paramyxoviruses such as parainfluenza virus type 3 (PIV3) [16,17]. Soon after the discovery of hMPV, MacPhail * et al.*, (2004) first tested the permissiveness of hMPV in Syrian golden hamsters [14]. In their study, they compared the replication of hMPV in four rodent models (mice, cotton rats, hamsters and ferrets) and two primate species, rhesus macaques and African green monkeys (AGMs). It was found that hamsters, ferrets and AGMs supported hMPV replication efficiently and produced high levels of hMPV-specific neutralizing antibody. Although no clinical signs were observed, hMPV infection caused pulmonary histological changes [14]. Using hamster as an animal model, it was found that animals immunized with hMPV from subgroups A (strain NL/1/00) and B (strain NL/1/99) can provide cross-challenge protection against homologous and heterologous hMPV infection [14]. In addition, several other groups also found that Syrian golden hamsters support the replication of various hMPV strains and thus are a useful model to evaluate the pathogenesis of hMPV strain *in vivo* and the efficacy of vaccine candidates [15,16,18,19,20,21].

Because cotton rats share certain similarities with humans upon respiratory tract infection, they were considered a preferred model for pediatric respiratory tract pathogens, such as measles, PIV3 and RSV [22,23,24,25,26,27]. In 2004, the first experiment using cotton rats to study hMPV infection was reported by MacPhail *et al.* In that study, less than 60 PFU/g of virus was detected in lung tissue and less than 50 PFU/g of virus was detected in nasal turbinate when cotton rats were inoculated with 6 log_10 _PFU of hMPV [14]. Thus, it was concluded that cotton rat is not a permissive model for hMPV replication. In contrast, Williams *et al.* reported that hMPV replicates efficiently in cotton rats [15]. Specifically, viral titer in the nasal turbinates (day 2) and lung tissues (day 4) reached 4.75 and 5.26 log_10_ PFU/g, respectively, when cotton rats were inoculated with 5 log_10_ PFU of hMPV strain TN/96-12. Wyde *et al.* (2005) reported that cotton rats support efficient replication for two hMPV subtype A strains and one subtype B strain [28]. The two groups showed that cotton rat is a good model to evaluate the efficacy of hMPV vaccine candidates [15,28,29,30]. Thus, cotton rats are a highly permissive model for hMPV infection. The reason for these controversial results observed by MacPhail *et al.* (2004), Williams *et al.* (2005), and Wyde *et al.* (2005) is unknown. Probably, it is due to the differences in hMPV strains, methodology, and environmental factors in the animal experiments.

Since results from the small animal studies are inconsistent and conflicting, it is necessary to reevaluate the permissiveness of these animal models for hMPV infection. This approach will allow us to identify a best small animal model to evaluate the safety and efficacy of hMPV vaccine candidates and therapeutic agents. In this study, we directly compared the replication of hMPV in the upper and lower respiratory tract in three animal models, mice, hamsters and cotton rats. Under our experimental conditions, we found that (i) BALB/c mice were not permissive for hMPV infection; (ii) hamsters supported viral replication in upper respiratory tract but showed limited replication in lower respiratory tract; and (iii) cotton rats supported efficient viral replication in both the upper and lower respiratory tract, indicating that cotton rats are a robust animal model for hMPV.

## 2. Results

### 2.1. Recovery of rhMPV with a Trypsin-Independent F Cleavage Site (rhMPV-F)

To improve the growth of hMPV, the cleavage site of fusion (F) protein of hMPV NL/1/100 was modified. Specifically, the ^99^RQSR^102^ motif in F cleavage site was mutated to ^99^RRRR^102^ in an infectious clone of hMPV NL/1/100, and the recombinant hMPV carrying the F cleavage site (rhMPV-F) was recovered using a procedure described previously [31]. As expected, rhMPV-F displayed a trypsin-independent growth pattern in LLC-MK2 cells or Vero-E6 cells. The entire genome of rhMPV-F was sequenced to confirm the presence of the desired mutations in the F cleavage and no additional mutations in viral genome. Recombinant rhMPV-F was grown in LLC-MK2 cells and used for all animal studies.

### 2.2. Replication of rhMPV-F in Mice

BALB/c mice have previously been used to study live attenuated RSV vaccine candidates [32]. Several researchers reported that mouse is a permissive model for hMPV [12,13]. However, others reported that hMPV replicates poorly in mice [14,15]. We first aimed to examine the infectivity of hMPV in mice. Briefly, mice were inoculated intranasally with 6.5 log_10_ PFU of rhMPV-F, and weight loss and presence of respiratory signs were monitored daily. No respiratory symptoms such as cough, increased mucus production, rhinitis, and heavy breathing were observed in the animals infected with rhMPV. No significant change in body weight was observed in rhMPV-F infected mice compared to mock infected control group (data not shown). No mortality, ruffled fur, or behavior changes were observed.

At day 4 post-inoculation, mice were sacrificed, and viral titer in the nasal turbinates and lungs was determined by an immunostaining assay on Vero-E6 cells. None of animals had detectable virus in nasal turbinate or lung tissues. To determine the presence of viral RNA in the lung, a one-step RT-PCR targeting the conserved region VI (CR VI) of hMPV L gene was also performed. No viral RNA was detected by RT-PCR (data not shown). Histological examination showed no significant changes compared with uninfected control. These observations showed that BALB/c mice are not a permissive animal model for rhMPV-F infection.

### 2.3. Replication of rhMPV-F in Syrian Golden Hamsters

**Figure 1:**
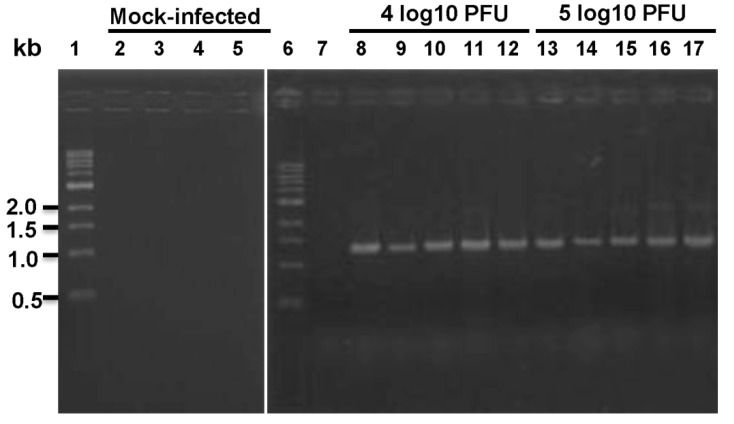
RT-PCR detection of viral RNA in lungs from hamsters inoculated with rhMPV-F. Lanes 1 and 6, 1kb DNA ladder; lanes 2–5 and 7, mock infected; lanes 8–12, hamsters infected by 4 log_10_ PFU of rhMPV-F; lanes 13–17, hamsters infected by 5 log_10_ PFU of rhMPV-F.

Syrian golden hamsters have been used to evaluate PIV3 vaccine candidates [16,33]. Several studies have reported that hamsters are more permissive to hMPV infection than mice [14,15]. In our first study, fifteen Syrian golden hamsters were randomly divided into groups of five animals. Group 2 and 3 were infected with 4 and 5 log_10_ PFU of rhMPV-F, respectively. Hamsters in group 1 served as mock infected control. No body weight loss or clinical signs were observed after virus inoculation. At day 4 post-infection, hamsters were euthanized and virus titers in nasal turbinates and lungs were measured by an immunostaining assay. The average virus titers in nasal turbinates were 4.43 and 4.60 log_10_ PFU, respectively. However, no infectious virus was detected in lung tissues of hamsters inoculated with either dose (Table 1). Since no infectious virus was found in hamster lungs, we sought to determine if viral RNA is present. Total RNA was extracted from lung tissue homogenates and RT-PCR was performed to target the conserved region VI (CR-VI) of the L gene. Although no infectious virus could be recovered, viral RNA was present in hamster lungs from both infected groups (Figure 1). To further test the role of the inoculation level, we used a higher dose of virus in a second experiment. Ten Syrian golden hamsters were randomly divided into two groups. Hamsters in group 1 were mock inoculated with DMEM and group 2 was inoculated intranasally with 6.5 log_10_ PFU of rhMPV-F. No significant weight change was observed between the hMPV-infected group and mock infected control (*p* > 0.05) (data not shown). At day 4 post-infection, the average titers in nasal turbinates and lungs were 4.29 and 2.88 log_10_ PFU, respectively.

**Figure 2:**
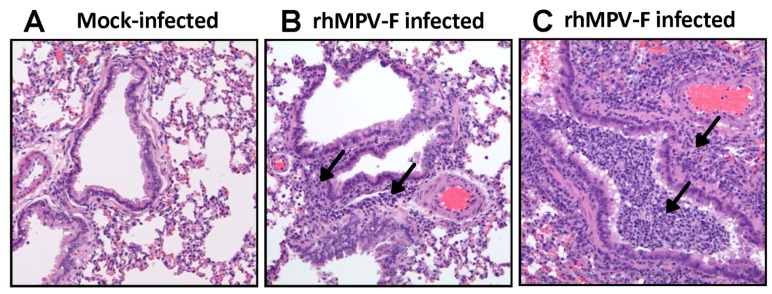
Histological changes in lungs of hamsters infected by rhMPV-F. A: Lungs from mock infected hamsters. B–C: Lungs from hamsters infected by rhMPV-F with a dose of 6.5 log_10 _PFU. Arrows in B indicate lymphoid infiltrates, and arrows in C show severe infiltrates with prominent acute inflammation involving a bronchiole and the adjacent parenchyma.

**Figure 3:**
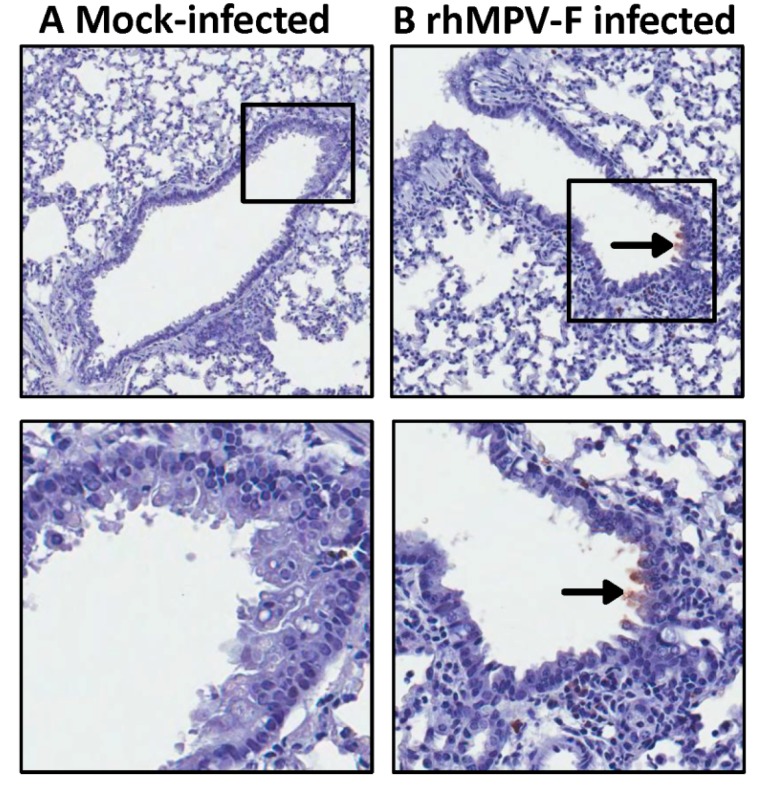
Immunohistochemical staining of lung tissues from hamsters infected by rhMPV-F. A: Lung from mock infected hamsters. Lungs were harvested at day 4 after mock infection. No hMPV antigen was detected. B: Lungs from hamsters infected by 6.5 log_10 _PFU of rhMPV-F and were sacrificed at day 4 post infection. A small amount of hMPV antigens were detected in the bronchial epithelial cells (indicated by arrows).

Subsequently, lung histology of hamsters infected with 6.5 log_10 _PFU of rhMPV-F was examined. It was found that lungs from rhMPV-F-infected hamsters displayed mild histological changes including lymphocytic infiltrates around bronchovascular bundles with extension into interlobular septae, interstitium and venules as well as acute inflammation involving bronchioles and alveolar parenchyma (Figure 2). These results showed that hamsters are permissive for rhMPV-F. However, rhMPV-F replicates in the upper respiratory tract but much less efficiently in the lower respiratory tract under our experimental conditions. To determine whether hMPV antigens were expressed, lung tissue sections were subjected to immunohistochemistry (IHC) analysis using a monoclonal antibody against hMPV matrix protein. As shown in Figure 3, no hMPV antigens were detected in mock infected lungs, and a small amount of antigens were found in bronchial epithelial cells.

**Table 1:** Replication of rhMPV-F in BALB/c mice and hamsters.

				Nasal turbinate		Lung	
Animals	Inoculum (log_10_ PFU)	Numbers of animals	Day ofharvest	% of infected animals	Mean titer(log_10_ PFU/ml)	% of infected animals	Mean titer (log_10_ PFU/g)
Mice	0	5	4	0	ND	0	ND *
Mice	6.5	5	4	0	ND	0	ND
Hamsters	0	5	4	0	ND	0	ND
Hamsters	4	5	4	100	4.43 ± 0.21	0	ND
Hamsters	5	5	4	100	4.61 ± 0.21	0	ND
Hamsters	6.5	5	4	100	4.29 ± 0.48	100	2.88 ± 0.04

*ND: Not detected

### 2.4. Replication of rhMPV-F in Cotton Rats

In experiment 1, we sought to determine if cotton rats are permissive for rhMPV-F infection. An experiment was designed to determine the optimal infection dose and best time to harvest nasal turbinates and lungs (Table 5). Twenty animals were divided into five groups. The first three groups were intranasally inoculated with 5 log_10_ PFU of rhMPV-F and terminated on 3, 4, and 5 days post infection. The other two groups were infected with 4 and 6 log_10_ PFU of virus, respectively, and terminated on 4 days post infection. No body weight loss or clinical signs were observed after virus inoculation (data not shown). The average virus titer in lung tissues of animals infected with 5 log_10_ PFU and harvested on day 3, 4, and 5 post infection were 4.43, 5.05, and 4.87 log_10_ PFU/g, respectively (Table 2). The average virus titer in lung tissues from cotton rats infected with 4 and 6 log_10_ PFU of virus harvested 4 days post-infection were 4.43 and 4.87 log_10_ PFU/g, respectively (Table 2). To test the viral replication in the upper respiratory tract, we performed nasal washes to isolate rhMPV-F in the nasal turbinate. No virus was detected in the nasal wash samples in the 5 log_10_ PFU group at day 3 post-infection. In the other groups the mean virus titers in the nasal wash were much lower than those in lungs, ranging from to 1.66 to 2 log_10_ PFU/ml. This experiment showed that rhMPV-F replicated efficiently in lungs of cotton rats.

**Table 2:** Replication of rhMPV-F in upper and lower respiratory tract in cotton rats。

				Nasal titer		Lung titer	
Experiment No.	Inoculum (log_10_PFU)	Numbers of animals	Day of termination	% of infected animals	Mean titer (log_10_PFU/g)	% of infected animals	Mean titer (log_10_PFU/g)
Experiment 1	5	4	3	0	ND	100	4.43 ± 0.27
	5	4	4	25	2	100	5.05 ± 0.47
	5	4	5	50	1.94	100	4.87 ± 0.40
	6	4	4	50	1.66	100	5.79 ± 0.12
	4	4	4	25	1.7	100	3.58 ± 0.51
Experiment 2	0	5	4	0	ND	0	ND
	5	5	4	100	5.20 ± 0.50	100	4.10 ± 0.30

Note: In experiment 1, nasal wash samples were used for virus titration. In experiment 2, the nasal turbinates were homogenized and used for virus titration. ND: not detected.

In experiment 2, we repeated the cotton rat experiment by inoculating 5 log_10_ PFU of rhMPV-F intranasally, and cotton rats were terminated at day 4 post-infection. In this experiment, both nasal turbinates and lungs were homogenized for virus recovery. As shown in Table 2, 5.20 and 4.10 log_10 _PFU/g of rhMPV-F were found in nasal turbinates and lungs, respectively. Thus, compared to nasal wash, homogenization of nasal turbinates significantly increased the efficiency of virus recovery. These results demonstrated that rhMPV-F replicated efficiently in both upper and lower respiratory tract.

**Figure 4:**
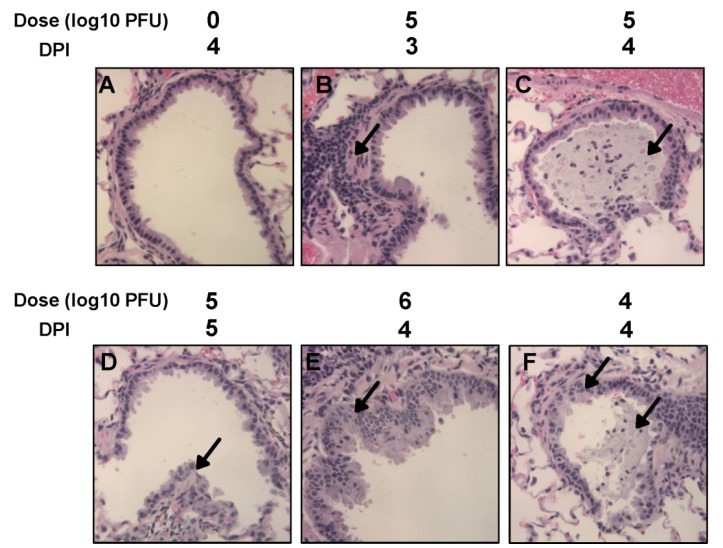
Histological changes in lungs of cotton rats infected by rhMPV-F. A: Lungs from mock infected cotton rats harvested at day 4. B, C, and D: Lungs from cotton rats intranasally infected by 5 log_10 _PFU of rhMPV-F and harvested at days 3, 4, and 5 post-infection, respectively. Arrow indicates histopathological changes including lymphoid infiltrates, bronchial luminal exudate, and airway remodeling. E-F: Lungs from cotton rats intranasally infected with 6 or 4 log_10 _PFU of rhMPV-F, respectively, and harvested at day 4 postinfection. Arrows indicate histopathological changes including lymphoid infiltrates, bronchial luminal exudate, and airway remodeling.

Histological examination found that infected lungs exhibited mild to moderate pathological changes including peribronchial and perivascular inflammation, airway remodeling, as well as interstitial pneumonia and increased bronchial exudate containing epithelial cell debris, neutrophils, macrophages (Figure 4).

**Figure 5:**
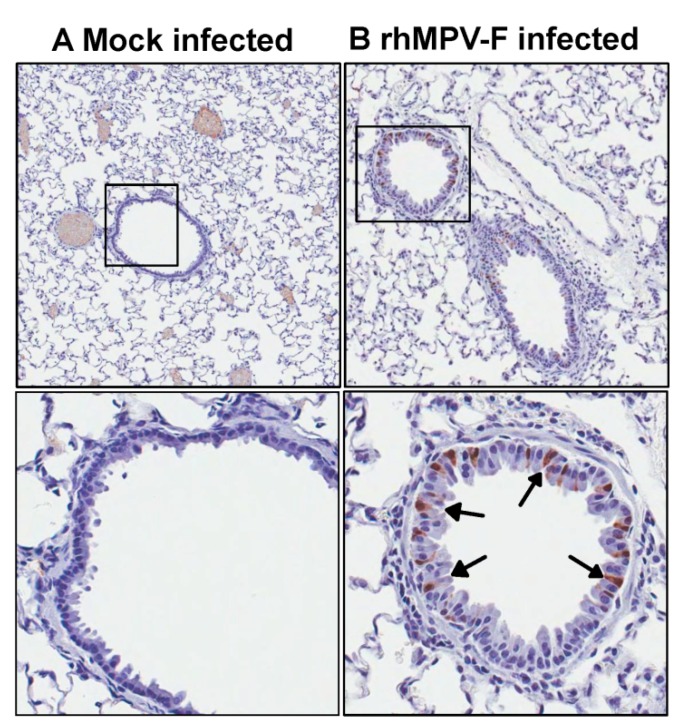
Immunohistochemical staining of lung tissues from cotton rats infected by rhMPV-F. A: Lungs from mock infected cotton rats. Lungs were harvested at day 4 after mock infection. No hMPV antigen was detected. B: Lungs from cotton rats infected by 6 log_10 _PFU of rhMPV-F and were sacrificed at day 4 post infection. Arrows indicate hMPV antigen in the infected bronchial epithelial cells.

To further confirm the replication of hMPV-F, lung tissue sections were subjected to immunohistochemistry (IHC) analysis using a monoclonal antibody against hMPV matrix protein. hMPV antigens were found in bronchial epithelial cells in a discontinuous pattern. In addition, luminal exudates including some visible macrophage cells were also positive for hMPV antigen (Figure 5). Collectively, these experiments demonstrated that (i) rhMPV-F replicates efficiently in nasal turbinates and lungs of cotton rats; (ii) rhMPV-F causes histological changes in lungs; and (iii) a large amount of rhMPV antigens was expressed in bronchial epithelial cells. Therefore, the cotton rat is a robust animal model for rhMPV-F infection.

### 2.5. Protective Immunity and Antibody Response in Cotton Rats

Next, we determined whether cotton rats can serve as an animal model to determine the protective immunity against rhMPV-F. Briefly, rhMPV-F was inoculated intranasally into cotton rats, and serum samples were collected weekly for the detection of humoral immune response. At week 4 post-inoculation, animals were challenged with 6 log_10_ PFU of rhMPV-F. At day 4 post-challenge, all the animals were sacrificed and nasal turbinates and lung samples were collected for virus detection and pathological examination.

#### 2.5.1. Serum-Virus Neutralizing Antibody

**Figure 6:**
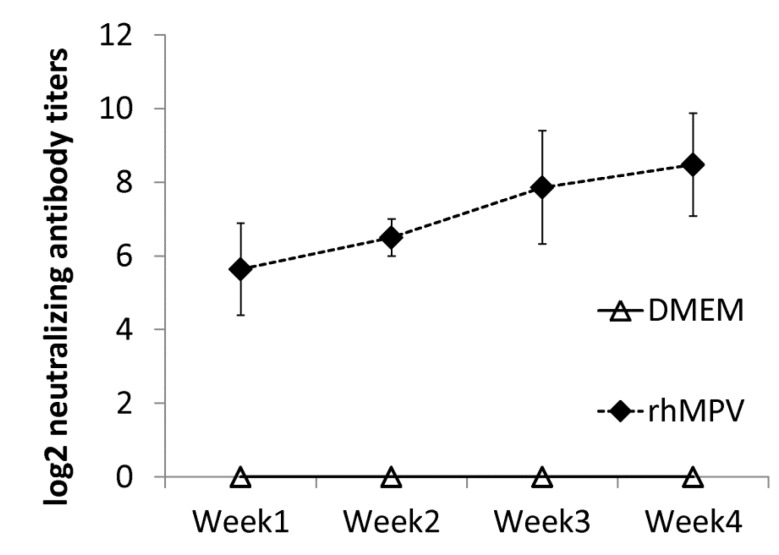
Serum neutralizing antibody titers in cotton rats vaccinated with rhMPV-F. Cotton rats were immunized by rhMPV-F intranasally at a dose of 5.3 log_10_ PFU per rat. Serum samples were collected from each cotton rat weekly after immunization. Serum neutralizing antibodies were determined using plaque reduction neutralization assay. The antibody titers were calculated as 50% plaque reduction titer.

Serum antibody was determined by a plaque reduction neutralization assay. As shown in Figure 6, rhMPV-F elicited high levels of neutralizing antibody in cotton rats. Antibody was detectable at week 1 post-immunization and gradually increased during weeks 2–4. In contrast, no hMPV-specific antibody was detected in the unvaccinated control. This result demonstrated that rhMPV-F was capable of triggering high levels of neutralizing antibody.

#### 2.5.2. Protective Immunity Against Viral Replication in Upper And Lower Respiratory Tracts in Cotton Rats

Viral replication in the nasal turbinate and lungs of cotton rats after challenge was determined by virus titration using an immunostaining assay. As shown in Table 3, cotton rats vaccinated with wildtype rhMPV-F did not have any detectable infectious virus particles in either the nasal turbinate or lungs after challenge with rhMPV-F. In contrast, unvaccinated challenge controls had average titers of 5.65 and 4.76 log_10_ PFU/g in the nasal turbinate and lung, respectively. These results demonstrated that rhMPV-F immunization provided complete protection against viral replication in both upper and lower respiratory tracts after challenge with wildtype rhMPV-F. 

**Table 3:** Immunogenicity of rhMPV-F in cotton rats.

Groups	Number of animals	Nasal Turbinate		Lung		
		% of infected animals	Mean titer (log_10_ PFU/g)	% of infected animals	Mean titer	
					log_10_ PFU/g	(log_10_ genomic RNA copies/g)
DMEMC	5	100	5.59 ± 0.49		100	4.76 ± 0.29
rhMPV-F	5	0	ND		0	ND
DMEM	5	0	ND		0	ND

Note: DMEMC indicates that cotton rats were inocuated with DMEM and challenged with rhMPV-F. DMEM indicates that cotton rats were inoculated with DMEM and served as uninfected controls. rhMPV-F indicate cotton rats were immunized with rhMPV-F and challenged with rhMPV-F. ND: not detected.

#### 2.5.3. Pulmonary Histopathology

**Figure 7:**
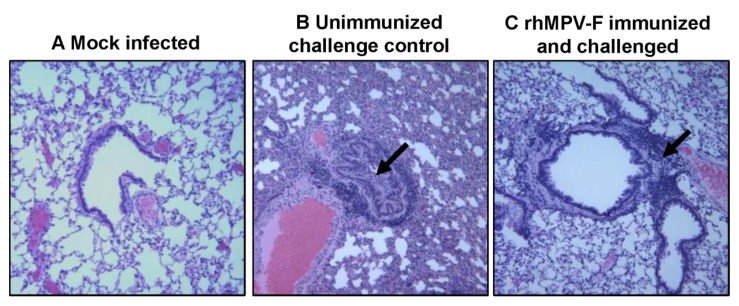
Lung histological changes in cotton rat immunogenicity and protection study. **A**: Lungs from mock infected cotton rats. **B**: Lungs from unimmunized challenge control group. Arrows indicate significant histological changes including interstitial pneumonia, lymphoid infiltrates, bronchial luminal exudate, and airway remodelling. **C**: Lungs from cotton rats immunized by rhMPV-F and challenged with the same virus. Significantly less histological changes (indicated by arrow) were observed in panels C compared to panel B. Minor histological changes but no enhanced lung damages were observed in panel C.

After challenge, lung histology was evaluated for each cotton rat. As expected, unvaccinated challenged controls had moderate pulmonary histological changes characterized by interstitial pneumonia, mononuclear cell infiltration, and edematous thickening of the bronchial submucosa (Figure 7). In contrast, significantly fewer histological changes were found in the lungs of cotton rats vaccinated with rhMPV-F (Table 4). No enhanced lung damage was observed in vaccinated groups. No histologic changes were found in uninfected controls. These results demonstrate that rhMPV-F immunization provided protection against lung damage from virulent challenge.

**Table 4:** Pulmonary histological changes in cotton rats infected by rhMPV-F.

Groups	Interstitial pneumonia	Bronchial epithelium change	Bronchial exudate	Peribronchial/perivascular mononuclear inflammation
DMEMC	1.60	1.0	0.5	1.75
rhMPV-F	0.60	0.5	0.2	1.4
DMEM	0	0	0	0

Note: The severity of lung histology (Interstitial pneumonia, Bronchial epithelium change, Bronchial exudate, and mononuclear inflammation) was scored for each lung tissues. Average score for each group is shown. 0 = no change; 1 = mild change; 2 = moderate change; and 3 = severe change.

#### 2.5.4. Viral antigen Distribution after Challenge

**Figure 8:**
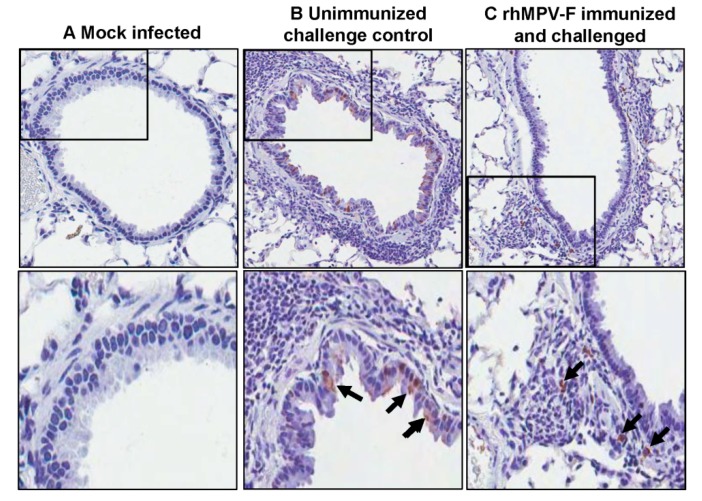
Immunohistochemical (IHC) staining of lungs of cotton rats. (**A**) Lungs from mock infected cotton rats. (**B**) Lungs from unimmunized challenge control group. A large number of hMPV antigen-positive cells were detected at the luminal surfaces of bronchial epithelial cells. (**C**) Lungs from cotton rats immunized by rhMPV-F and challenged with the same virus. hMPV antigens were found inside the bronchial tissue, but not on the luminal surface of the bronchial epithelial cells.

For lung tissues from unvaccinated challenged controls, a large amount of viral antigen was found at the luminal surface of the bronchial epithelial cells. Interestingly, lung tissues from vaccinated animals exhibited a different antigen distribution pattern (Figure 8). A significant amount of antigens were found inside of the bronchial tissue, but not on the luminal surface of the bronchial epithelial cells. No antigen was found in the naive group.

## 3. Discussion

A robust animal model is essential to evaluate the safety and efficacy of hMPV vaccine candidates. We compared the replication and pathogenesis of rhMPV-F strain NL/1/00 in three rodent models, BALB/c mice (*Mus musculus*), Syrian golden hamsters (*Mesocricetus auratus*), and cotton rats (*Sigmodon hispidus*). We found that the cotton rat is the best model for hMPV-F whereas hamster is less permissive and mouse is not permissive.

Currently, many controversial results have been reported using the mouse as an animal model for hMPV infection. It was first reported that hMPV causes clinical signs and breathing difficulties in BALB/c mice [12]. In addition, the authors observed a biphasic growth kinetics for hMPV in lungs of BALB/c mice with a high viral (7–8 log_10 _PFU/g lung tissue), which has not been observed for hMPV replication in any other animal models. Another group reported that hMPV infection causes modest mononuclear cell infiltration in the interstitium, airway remodeling, increased mucus production and bronchial infiltration in lungs [13]. However, many other studies found that hMPV does not cause any clinical signs or viral replication in BALB/c mice [14,15]. It is possible that the ability of hMPV replication may vary in different strains of mice (*Mus musculus*). However, it was found that replication of hMPV in mice strains of 129, AKR, BALB/c, C3H, C57BL/10, CBA, DBA/1, DBA/2, and SJL were extremely low, ranging from undetectable to 2.93 log_10_ PFU/g lung tissue [14,15]. In our study, we were not able to detect any infectious virus or viral RNA from nasal turbinate and lung tissue of any animal. Our results support the conclusion that BALB/c mice are not permissive for hMPV infection.

Syrian golden hamsters were first reported to be permissive for hMPV infection by MacPhail *et al.* in 2004 [14]. In that study, hamsters were inoculated intranasally with a 0.1 ml volume containing 1.3 ×10^6^ PFU of hMPV strain NL/1/00. The authors found 5.3 and 4.3 log_10_ PFU/g tissue of virus in nasal turbinates and lung, respectively, at day 4 post-inoculation. Other studies also found that Syrian golden hamsters are permissive for hMPV replication in the upper and lower respiratory tract, but the efficiency of viral replication is lower than that in cotton rats [15]. In our study, we compared the replication of three different doses of rhMPV-F (derived from strain NL/1/00) in Syrian golden hamsters. At high dose (6.5 log_10_ PFU), 4.29 and 2.88 log_10_ PFU of virus was detected in nasal turbinates and lungs, respectively. In contrast, 4–5 log_10_ PFU virus was found in nasal turbinates but no infectious virus was detected in lungs when lower doses (5 and 4 log_10_ PFU) of virus were used for inoculation. Although infectious rhMPV-F was not detected in lungs, viral RNA was present in hamster lungs infected by both doses of rhMPV-F. The mechanism of this viral RNA presence is not clear yet. It may result from limited viral replication in lung or residual virus particles from the inoculum. Alternatively, RT-PCR may be more sensitive than virus titration. In addition, rhMPV-F infection caused significant histological changes in lungs of hamsters. Overall, the replication efficiency in lungs of hamsters under our experimental conditions was lower than those previously reported [14,15]. Perhaps, this difference may result from different virus strains, different inbred hamster strains used for infection, and/or environmental factors (such as humidity and temperature) in animal facilities. It should be mentioned that our study used the same hMPV strain (NL/1/00) as MacPhail’s hamster study in 2004 except that we modified the F cleavage site of hMPV NL/1/00. It is possible that this modification may alter viral replication in hamsters. Overall, our results support the conclusion that hamsters are a permissive animal model for rhMPV-F. However, hamsters do not appear to support efficient replication of rhMPV-F in lower respiratory tract under our experimental conditions.

The cotton rat *S.* *hispidus* is a small rodent model susceptible to a large variety of human pathogens [34]. This model has been found to be the best model for respiratory viruses. Previously, it was found that permissiveness of cotton rats to infection with human respiratory syncytial virus (hRSV) was over 100-fold higher than that of mice. In fact, cotton rats have not only been used as an animal model to study the pathogenesis and efficacy of hRSV vaccine, but also for evaluating the efficacy and safety of prophylactic antibody RespiGam and Synagis treatment for severe hRSV disease. The cotton rat model also accurately predicted the effective dose of the drug currently being used in human infants. In recent years, many tools and reagents (such as cytokines, chemokines, cell surface markers, and regulatory molecules) have been developed for cotton rats that accelerated the use of this model for human pathogens.

The first hMPV infection in cotton rats was done in 2004 by MacPhail [14]. Five cotton rats were intranasally inoculated with 6.11 log_10_ PFU of hMPV strain NL/1/00. Less than 50 and 60 PFU/g tissue of virus was detected in nasal turbinate and lung in cotton rats, respectively. In contrast, another group determined the kinetics of hMPV replication in cotton rats and found that hMPV replicates efficiently in cotton rats [15]. In their study, cotton rats were inoculated with 5 log_10_ PFU of hMPV and were sacrificed at 2, 4, 6, 8, 10, or 14 days post infection. It was found that the replication of hMPV in the lung tissues peaked on day 4 post infection at a mean titer of 5.26 log_10_ PFU/g and declined gradually. Virus was not detected in the lung after day 6. Similarly, several other groups found that cotton rats are permissive for hMPV replication in nose and lungs, with a peak titer of 3.6 log_10_ PFU/g in the nose and 4.4 log_10_ PFU/g in the lung on day 4 post-infection [28,29,30,35]. In our study, we compared rhMPV-F replication in cotton rats using three different inoculation doses at different time points. We found that rhMPV-F replicates efficiently in the lungs of cotton rats, and that the level of output virus was dependent on the dose of input virus. At an inoculation dose of 4 log_10_ PFU per rat, 3.58 log_10_ PFU/g of virus was found in lungs. When the inoculation dose was increased to 5 or 6 log_10 _PFU per rat, virus titer in lungs increased to 5.04 and 5.79 log_10_ PFU/g, respectively (*p* < 0.05). Day 4 post-infection appears to be the best time to terminate the study as the viral titer in lungs at this time point has peaked. The replication titer on day 4 post-infection is significantly higher that on day 3 (*p* < 0.05). However, no significant difference was observed between the titers on days 4 and 5 *(p* > 0.05). Furthermore, we demonstrated that rhMPV-F caused significant histological changes in lungs although no respiratory symptoms were observed. Our results were consistent with those previously reported [15,28]. Also, it should be pointed out that we recovered less infectious virus using nasal washing. However, we found that efficiency of virus recovery significantly increased when we homogenized the nasal turbinate (Table 2). It should be noted that our study used the same hMPV strain (NL/1/00) as MacPhail’s cotton study which showed that cotton rat is not permissive for hMPV infection. Again, we cannot exclude the possibility that the modification of F cleavage site of hMPV strain NL/1/00 may alter viral replication in cotton rats. Given the fact that cotton rats support efficient replication of a number of other hMPV strains from both subgroups A and B observed by several other researchers [13,15,28], we concluded that cotton rats is a robust model for hMPV replication. These results, are also consistent with previous observations on RSV, PIV3, and measles virus, suggest that the cotton rat is an ideal animal model to study human paramyxoviruses.

An ideal animal model should also accurately evaluate the safety and efficacy of vaccine candidates and therapeutic agents. One concern for paramyxovirus vaccine development is the risk of enhanced disease. In the 1960s, a formalin-inactivated RSV (FI-RSV) vaccine trial led to more severe disease after subsequent re-exposure to wildtype virus infection, resulting in hospitalization of 80% of vaccine recipients and two deaths. In fact, cotton rat has been used as a model to study the mechanism of the enhanced lung disease. Specifically, it was found that RSV caused enhanced pulmonary pathology in cotton rats that had been vaccinated with FI-RSV, followed by reinfection of RSV [24,36]. Similar vaccine enhanced disease as reproduced in cotton rats for FI-PIV3 and FI-hMPV [37,38]. These similarities suggest that cotton rats can be used as a model to evaluate the safety and efficacy of paramyxovirus vaccines. In this study, cotton rats were immunized with rhMPV-F and challenged with rhMPV-F. All immunized animals produced a high level of serum neutralizing antibody response and were completely protected from viral replication in the upper and lower respiratory tract after challenge with rhMPV-F. In addition, no enhanced pulmonary disease was observed and the immunized animals were protected from histologic changes in the lungs. Interestingly, a large amount of viral antigen was found at the luminal surfaces of the epithelial cells of the bronchi in unvaccinated and challenged groups. This is consistent with the previous observation that viral antigens were localized almost exclusively at the apical surfaces of ciliated respiratory epithelial cells [39]. However, viral antigens were distributed inside but not on the luminal surface of the bronchi in vaccinated challenged groups. One possibility is that alveolar macrophages and/or dendritic cells were activated by vaccination with live vaccine antigens, and upon virus challenge migrated to the respiratory surfaces to capture viral antigens. It is also possible that alveolar mucosal antibody binds and neutralizes the virus, which was captured by immune cells. Further study is needed to elucidate the mechanism of viral clearance. Cotton rats showed protective immunity and antibody response against hMPV, and thus are an excellent animal model to evaluate vaccine candidates.

## 4. Experimental Section

### 4.1. Cell Lines

LLC-MK2 (ATCC No. CCL-7) cells were maintained in Opti-MEM (Life Technologies, Bethesda, MD, USA) supplemented with 2% fetal bovine serum (FBS). Vero E6 cells (ATCC No. CRL-1586) was grown in Dulbecco’s modified Eagle's medium (DMEM; Life Technologies) supplemented with 10% FBS.

### 4.2. Recovery and Characterization of Recombinant hMPV-F

An infectious cDNA clone of hMPV lineage A strain NL/1/100 was kindly provided by Dr. Ron A. M. Fouchier at Department of Virology, Erasmus Medical Center, Rotterdam, The Netherlands. The enhance the growth of the virus, the F cleavage site (99-RQSR-102) in the full-length genome of wild type hMPV NL/1/100 was mutated into 99-RRRR-102 using the QuikChange Site-Directed Mutagenesis Kit (Strategene, La Jolla, CA, USA) with the following primers: forward, 5'-GAGAGGAGCAAATTGAAAATCCCAGACGACGTAGATTCGTTCTAGGAGCAATAGC-3'; reverse, 5'-GCTATTGCTCCTAGAACGAATCTACGTCGTCTGGGATTTTCAATTTGCTCCTCTC-3'. The resultant plasmid is designated as phMPV-F. Recombinant hMPV carrying the F cleavage site mutation (rhMPV-F) was recovered using a reverse genetics system [31]. Briefly, rhMPV-F was recovered by cotransfection of a plasmid encoding the full-length genomic cDNA of hMPV NL/1/00 (phMPV-F) and support plasmids encoding viral N (pCITE-N), P (pCITE P), L (pCITE-L), and M2-1 (pCITE-M2-1) proteins into BHK.SR19T7pac cells (kindly provided by Apath LLC, Brooklyn, NY, USA) which express stably the T7 RNA polymerase. Six days posttransfection, the cells were subjected to three freeze-thaw cycles followed by centrifugation at 3000 × g for 10 min. The supernatant was used subsequently to infect LLC-MK2 cells. Cytopathic effect (CPE) was observed 5 days post-infection and the recovered viruses were amplified further in LLC-MK2 cells. Unlike wild type rhMPV which requires trypsin to grow, recombinant rhMPV-F exhibited trypsin-independent growth. Thus, rhMPV-F was propagated subsequently in LLC-MK2 cells in the absence of trypsin. Viral stocks were prepared at passage 4, and viral titer was determined by an immunostaining assay in Vero E6 cells.

### 4.3. Purification of Recombinant hMPV (rhMPV-F)

Recombinant rhMPV-F was purified and used in animal studies [40]. Briefly, LLC-MK2 cells were infected with rhMPV-F at MOI = 0.01 and incubated for 6 days at 37 °C and harvested by scraping. The cell suspension was clarified by low-speed centrifugation at 1500 rpm for 20 min at 4 °C in a Beckman Coulter Allegra 6R centrifuge. The supernatants were collected and the cell pellet was resuspended in 2 mL of DMEM and subjected to three freeze and thaw cycles. After centrifugation at 5000 rpm for 10 min, supernatants were collected and combined. The virus was pelleted by ultracentrifugation at 28,000 rpm in a Beckman Ty 50.2 rotor for 2 h. The virus pellet was resuspended in Opti-MEM and stored at −80 °C. Viral titer was determined by an immunostaining assay.

### 4.4. Replication of rhMPV-F in Mice

Ten four-week-old specific-pathogen-free (SPF) female BALB/c mice were purchased from Charles River Laboratories (Malvern, PA, USA). These animals were housed within ULAR facilities of The Ohio State University under approved Institutional Laboratory Animal Care and Use Committee (IACUC) guidelines. The animals were randomly divided into 2 groups (5 per group). Animals in group 1 were mock infected with Opti-MEM, and animals in group 2 were inoculated intranasally with 6.5 log_10 _PFU of rhMPV-F under isoflurane anesthesia. After inoculation, the animals were evaluated on a daily basis for weight loss and the presence of any respiratory symptoms of hMPV. At day 4 post-infection, animal were sacrificed and their nasal turbinates and lungs were removed for virus titration and pathological examination. (i) Virus titer in lung and nasal turbinate. Nasal turbinate and left lung from each animal were weighed and homologized in 1 ml of phosphate-buffered saline (PBS) using Dounce homogenizer. Viral titers were determined by an immunostaining assay. (ii) Total viral RNA was extracted and detected by RT-PCR. (iii) Pulmonary histology. Left lung from each mouse was fixed with 10% neutral buffered formalin for histology as described below.

### 4.5. Replication of rhMPV-F in Hamsters

Fifteen and ten four-week-old SPF female Golden Syrian Hamsters were purchased from Charles River Laboratories for two separate hamster studies. Animals were randomly divided into five animals per group. In the first experiment, hamsters in groups 2–3 were intranasally inoculated with 100 µL of Opti-MEM containing 4 and 5 log_10 _PFU of rhMPV-F, respectively. In the second experiment, hamsters in group 2 were infected with 6.5 log_10_ PFU of rhMPV-F. Animals in group 1 in both experiments were mock infected with 100 µL of Opti-MEM. After inoculation, the animals were evaluated on a daily basis for possible weight loss and the presence of any respiratory symptoms of hMPV-F. At day 4 post-infection, animal were sacrificed and their nasal turbinates and lungs were removed for virus titration and pathological examination as described.

### 4.6. Replication of rhMPV-F in Cotton Rats

In experiment 1, twenty SPF cotton rats were randomly divided into five groups (4 rats per group). These cotton rats were housed within ULAR facilities of The Ohio State University under approved animal use protocols. Each inoculation group was separately housed in rodent cages under BSL-2 conditions. Experimental design was summarized in Table 5. Rats were anaesthetized under isofluorane and infected with 0.1 mL of Opti-MEM containing different amounts of rhMPV-F. After inoculation, the animals were evaluated on a daily basis for the presence of any respiratory symptoms. At designated days post-infection, nasal washes were collected from each cotton rats. Briefly, 100 µL of PBS was dropped into nasal cavity of cotton rats, and the liquids were collected for virus titration. At designated days post-infection, cotton rats were sacrificed and their lungs were collected for virus isolation and histological analysis. In experiment 2, SPF cotton rats (Harlan Laboratories, Indianapolis, IN, USA) were randomly divided into 2 groups (5 rats per group). Cotton rats in group 1 were inoculated with DMEM and served as uninfected controls. Cotton rats in group 2 were inoculated with DMEM containing 5 log_10_ PFU of rhMPV-F. All the experimental procedures were identical to experiment 1 with the following changes. At day 4 post-infection, cotton rats were sacrificed, and their nasal turbinates and lungs were collected and homogenized for virus isolation.

**Table 5:** Experiment design of rhMPV-F replication study in cotton rats.

Dose (log_10_PFU)	Termination Time (day)		
	3	4	5
4.0		√	
5.0	√	√	√
6.0		√	

### 4.7. Immunogenicity of rhMPV-F in Cotton Rats

For the immunogenicity study, fifteen cotton rats (Harlan Laboratories, Indianapolis, IN) were randomly divided into 3 groups (5 rats per group). Rats in group 1 were mock infected with Opti-MEM as an uninfected control. Rats in group 2 were intranasally inoculated with 5.30 log_10_ PFU of rhMPV-F in 0.1 mL of Opti-MEM. Rats in group 3 were inoculated with DMEM and served as the unimmunized challenged control. After immunization, cotton rats were evaluated daily for mortality and the presence of any symptoms of hMPV infection. Blood samples were collected from each rat weekly by facial vein retro-orbital bleeding, and serum was isolated for neutralizing antibody detection. At week 4 post-immunization, rats in group 2 were challenged intranasally with rhMPV-F at a dose of 6 log_10_ PFU per rat. After challenge, the animals were evaluated twice every day for mortality and the presence of any symptoms of hMPV infection. At day 4 post-challenge, all rats from each group were euthanized by CO_2_ asphyxiation. The lungs and nasal turbinate from each rat were collected for virus isolation and histological evaluation. The immunogenicity of the hMPVs was evaluated using the following methods: (i) humoral immunity was determined by a virus-serum neutralization assay using an end-point dilution plaque reduction assay. (ii) Viral titer in the nasal turbinate and lungs was determined by an immunostaining plaque assay and viral genomic RNA was quantified by real-time RT-PCR. (iii) Pulmonary histopathology and viral antigen distribution was determined using the procedure described below. The protection was evaluated with respect to viral replication, antigen distribution, and pulmonary histopathology.

### 4.8. Pulmonary Histology

Right lung of each animal was removed, inflated and fixed by 4% formaldehyde (pH 7.0). Fixed tissues were embedded in paraffin, and sectioned at 5 microns. Slides were then stained with hematoxylin and eosin (H&E). The pulmonary histological changes were examined by three independent pathologists. Histological changes were scored based on the extent of inflammation (focal or diffuse), the pattern of inflammation (peribronchilolar, perivascular, interstitial, and alveolar), and the nature of the cells making up the infiltrate (neutrophils, eosinophils, lymphocytes, and macrophages).

### 4.9. Immunohistochemical Staining (IHC)

Right lung of animal were fixed in 10% formalin and embedded in paraffin. 5-micron sections were cut and placed onto positively charged slides. After deparafinization, sections were incubated with target retrieval solution (Dako, Carpinteria, CA, USA) for antigen retrieval. After blocking, a primary mouse anti-hMPV monoclonal antibody (Virostat, Portland, ME, USA) was incubated for 30 min at room temperature followed by incubation with a biotinylated horse anti-mouse secondary antibody (Vector Laboratories, Burlingame, CA, USA). Slides were further incubated with ABC Elite complex to probe biotin (Vector Laboratories) and slides were then developed using a 3,3'-diaminobenzidine (DAB) chromogen Kit (Dako) and hematoxylin used as a counterstain. Lung sections from hMPV infected and uninfected samples were used as positive and negative controls respectively.

### 4.10. Determination of Viral Titer in Lung and Nasal Turbinate

Virus titer was determined by an immunostaining assay. For animal experiments, nasal turbinate and the left lung from each animal were removed, weighed, and homogenized in 1 ml of phosphate-buffered saline (PBS) solution using Precellys 24 tissue homogenizer (Precellys, Bertin, MD, USA) following manufacturer’s recommendations. The homogenized tissues were clarified by centrifugation at 6000 rpm for 5 min at 4 °C. The presence of infectious virus was determined by an immunostaining plaque assay in Vero E6 cells. Briefly, Vero E6 cells were seeded in 24-well-plates and then infected with 0.2 ml of 10 fold serial dilutions of supernatant from homogenized tissues. After 1 adsorption, 0.5 ml of fresh culture medium containing 2% FBS and 0.75% methylcellulose (Sigma, St. Louis, MO, USA) was added to each well and incubated in humidified 5% CO_2_ incubator at 37 °C. At day 6 post-infection, the supernatant was removed and cells were fixed in a pre-chilled acetone: methanol solution (at the ratio of 3:2) at room temperature (RT) for 15 min. Cells were permeablized in a phosphate saline buffer (PBS) containing 0.4% Triton X-100 at RT for 10 min, and blocked at 37 °C for 1 hour using 1% bovine serum albumin (BSA) in PBS. The cells were then labeled with an anti-hMPV N protein primary monoclonal antibody (Millipore, Billerica, MA, USA) at dilution of 1:1000, followed by incubation with horseradish peroxidase (HRP)-labeled rabbit anti-mouse secondary antibody (Thermo Scientific, Waltham, MA, USA) at dilution of 1:5000. After incubation with AEC substrate chromogen (Sigma), positive cells were then visualized under the microscope. Viral titer was calculated as plaque forming unit (PFU) per gram tissue.

### 4.11. Reverse Transcription Polymerase Chain Reaction (RT-PCR)

Lung tissues from mice or hamsters infected by rhMPV-F were homogenized in 1 mL of PBS as described above. Total RNA was extracted from 200 µL of lung homogenate using an RNeasy mini-kit (Qiagen, Valencia, CA, USA) following the manufacturer's recommendation. RT-PCR was performed using a One Step RT-PCR kit (Qiagen) with two hMPV specific primers spanning the conserved region VI (CR VI) of the L gene,

hMPV-L-11759-Foward: 5'-TATATAGGGTTTAAGAATTGG-3'

hMPV-L-13199-Reverse: 5'-ATCATTTTTTACTTACAAGC-3'

For the cycling parameters, a holding stage at 50 °C was maintained for 30 min for reverse transcription. Temperature was raised to 95 °C to activate polymerase prior to cycling, followed by 35 cycles of 94 °C for 1 min for denaturation, 60 °C for 1 min for annealing, and 72 °C for 1 min for extension.

### 4.12. Quantification of hMPV genomic RNA by Real-Time RT-PCR

Viral genomic RNA copy in lung and nasal homogenate was determined by real-time RT-PCR. First strand cDNA was synthesized by SuperScriptase III (Life Technologies, Carlsbad, CA, USA) using the following primer targeting the hMPV leader sequence:

hMPV-leader-F-10:

5'-AAAACGCGTATAAATTAGATTCCAAAA-3'

A fragment covering partial leader sequence and partial N gene was quantified by SYBR Green real-time PCR (Life Technologies) using the following primers:

hMPV-leader-F-10:

5'-AAAACGCGTATAAATTAGATTCCAAAA-3'

hMPV-N-157-reverse:

5'-TGCAGTTGTTGTACCACATCTCTTT-3'

Real-time PCR was performed on a StepOne real-time PCR machine (Applied Biosystems, Foster City, CA, USA). PCR reaction and cycling parameters were set following the manufacturer’s recommendations. For cycling parameters, a holding stage at 95 °C was maintained for 10min to activate polymerase prior to cycling, followed by 50 cycles of 95 °C for 15 s for denaturation and 60 °C for 1 min for annealing and extension. Standard curves and StepOne Software v2.1 were used to quantify genomic RNA copies. Viral RNA was expressed as mean log_10_ genomic RNA copies per gram tissue ± standard deviation.

### 4.13. Determination of Neutralizing Antibody Against hMPV

hMPV-specific neutralizing antibody titers were determined using a plaque reduction neutralizing assay. Briefly, cotton rat sera were collected by retro-orbital bleed weekly until prior to challenge. The sera samples were heat inactivated at 56 °C for 30 min. Two-fold dilutions of serum samples were mixed with an equal volume of DMEM containing approximately 100 PFU/well rhMPV-F in a 96-well plate and incubated at room temperature for 1 h with constant rotation. The mixtures were then transferred to confluent Vero E6 cells in a 96-well plate in triplicate. After 1 h incubation at 37 °C, the virus-serum mixtures were removed and the cells were overlaid with 0.75% methylcellulose in DMEM and incubated for another 4 days before virus plaque titration as described previously. Plaques were counted and 50% plaque reduction titers were calculated as hMPV-specific neutralizing antibody titers.

### 4.14. Sequencing

Viral RNA was extracted from rhMPV-F using an RNeasy minikit (Qiagen) following the manufacturer’s recommendation. The full-length genome of rhMPV-F was amplified by six overlapping fragment by RT-PCR. The PCR products were purified and sequenced at The Ohio State University Plant Microbe Genetics Facility to confirm the presence of the designed mutation.

### 4.15. Statistical Analysis

Statistical analysis was performed by one-way multiple comparisons using SPSS 8.0 statistical analysis software (SPSS Inc., Chicago, IL, USA). A *p* value of <0.05 was considered statistically significant.

## 5. Conclusions

We compared the replication and pathogenesis of rhMPV-F in BALB/c mice, Syrian golden hamsters, and cotton rats. It was found that BALB/c mice are not permissive for rhMPV-F infection. In hamsters, rhMPV-F replicated efficiently in nasal turbinates but was restricted in lungs. Importantly, cotton rat is a robust small animal model for rhMPV. First, rhMPV-F replicated efficiently in both nasal turbinates and lungs of cotton rats when intranasally administered with three doses (4, 5, and 6 log_10_ PFU). Second, lungs of cotton rats infected by rhMPV-F developed histological changes including interstitial pneumonia, mononuclear cells infiltrates, and increased lumen exudates. Third, rhMPV-F antigens were expressed at the luminal surfaces of the bronchial epithelium cells in lungs. Finally, cotton rats infected by rhMPV-F developed protective immunity, thus can be used as a model to evaluate the safety and efficacy of vaccine candidates.
